# Differential Performance of the FilmArray Meningitis/Encephalitis Assay To Detect Bacterial and Viral Pathogens in Both Pediatric and Adult Populations

**DOI:** 10.1128/spectrum.02774-21

**Published:** 2022-04-11

**Authors:** Aurélie Schnuriger, Sophie Vimont, Alexandre Godmer, Joël Gozlan, Salah Gallah, Muriel Macé, Valérie Lalande, Kenda Saloum, Marine Perrier, Nicolas Veziris, Laurence Morand-Joubert

**Affiliations:** a Department of Virology, St Antoine – Tenon − Trousseau University Hospitals, Assistance Publique Hôpitaux de Paris, Paris, France; b Sorbonne Université, INSERM UMR_S 938, Centre de Recherche Saint-Antoine (CRSA), Paris, France; c Department of Bacteriology, Saint-Antoine Hospital, APHP.Sorbonne-Université, Paris, France; d Sorbonne Université, INSERM UMR S_1155, Tenon University Hospital, Paris, France; e Sorbonne Université, Centre d’Immunologie et des Maladies Infectieuses (Cimi-Paris), UMR 1135, Centre National de Référence des Mycobactéries, Paris, France; f Sorbonne Université, INSERM UMR_S 1136, Institut Pierre Louis d’Epidémiologie et de Santé Publique (iPLESP), Paris, France; University of Cincinnati

**Keywords:** meningitis/encephalitis, pediatric/adult population, performance, sensitivity/specificity, syndromic assay

## Abstract

Meningitis/encephalitis (ME) syndromic diagnostic assays can be applied for the rapid one-step detection of the most common pathogens in cerebrospinal fluid (CSF). However, the comprehensive performance of multiplex assays is still under evaluation. In our multisite university hospital of eastern Paris, France, ME syndromic testing has been gradually implemented since 2017 for patients with neurological symptoms presenting to an adult or pediatric emergency unit. We analyzed the results from the BioFire FilmArray ME panel versus standard routine bacteriology and virology techniques, together with CSF cytology and clinical data, over a 2.5-year period to compare the diagnostic accuracy of the FilmArray ME panel to that of the reference methods. In total, 1,744 CSF samples from 1,334 pediatric and 336 adult patients were analyzed. False-positive (mostly bacterial) and false-negative (mostly viral) cases were deciphered with the help of clinical data. The performance of the FilmArray ME panel in our study was better for bacterial detection (specificity >99%, sensitivity 100%) than viral detection (specificity >99%, sensitivity 75% for herpes simplex virus 1 [HSV-1] and 89% for enterovirus), our study being one of the largest, to date, concerning enteroviruses. The use of a threshold of 10 leukocytes/mm^3^ considerably increased the positive agreement between the results of the FilmArray ME panel and the clinical features, especially for bacterial pathogens, for which agreement increased from 58% to 87%, avoiding two-thirds of inappropriate testing. Based on this analysis, we propose an algorithm for the use of both syndromic and specific assays for the optimal management of suspected meningitis/encephalitis in adult and pediatric patients.

**IMPORTANCE** Based on our comparative analysis of performances of the diagnostic assays, we propose an algorithm for the use of both syndromic and specific assays, for an optimal care of the meningitis/encephalitis threat in adult and pediatric patients.

## INTRODUCTION

Infectious meningitis and encephalitis are potentially life-threatening diseases and expose patients to the risk of neurological sequelae if not rapidly diagnosed and treated. Bacterial, viral, or fungal agents can be involved and can share similar initial clinical presentations. However, the clinical evolution is closely related to the pathogen. Enterovirus (EV)-caused meningitis generally resolves spontaneously after 48 to 72 h, whereas herpes simplex virus (HSV) is capable of serious cerebral invasion in the absence of acyclovir treatment. Similarly, bacterial infections, generally originating from the ear, nose, and throat area, have the potential to cause septic meningitis, which can potentially evolve to meningoencephalitis, with a high risk of death in the absence of effective antibiotic treatment. Among young infants, rapidly invasive infection is feared and justifies urgent appropriate treatment. The diagnosis of meningitis or encephalitis first requires a lumbar puncture and the application of accurate pathogen-detection methods to cerebrospinal fluid (CSF), associated with prompt cytological analysis, to provide evidence of an increased leukocyte count. Meningitis/encephalitis (ME) syndromic detection assays have become available in recent years and cover the most common pathogens in a one-step rapid analysis validated on CSF (1). A timely diagnosis is obviously required in this ME setting. However, the true performance of such syndromic assays is still under evaluation, and disparate results have been reported (2). In our multisite university hospital in eastern Paris, France, ME syndromic testing has been gradually implemented since 2017 for adult and pediatric patients with neurological symptoms presenting to an emergency unit. This strategy was initially mostly driven by the need for a rapid response, especially for bacterial pathogens, because the local bacteriology units were moved to a single remote common site. The FilmArray ME multiplex PCR assay was chosen and first deployed in the emergency laboratories of the pediatric and then adult sites, followed by the centralized bacteriology laboratory. Our aim was to evaluate the diagnostic accuracy and performance of this syndromic assay relative to routine reference bacteriology and virology techniques for optimal pediatric and adult patient care.

## RESULTS

### Study population and pathogen detection.

**FIG 1:**
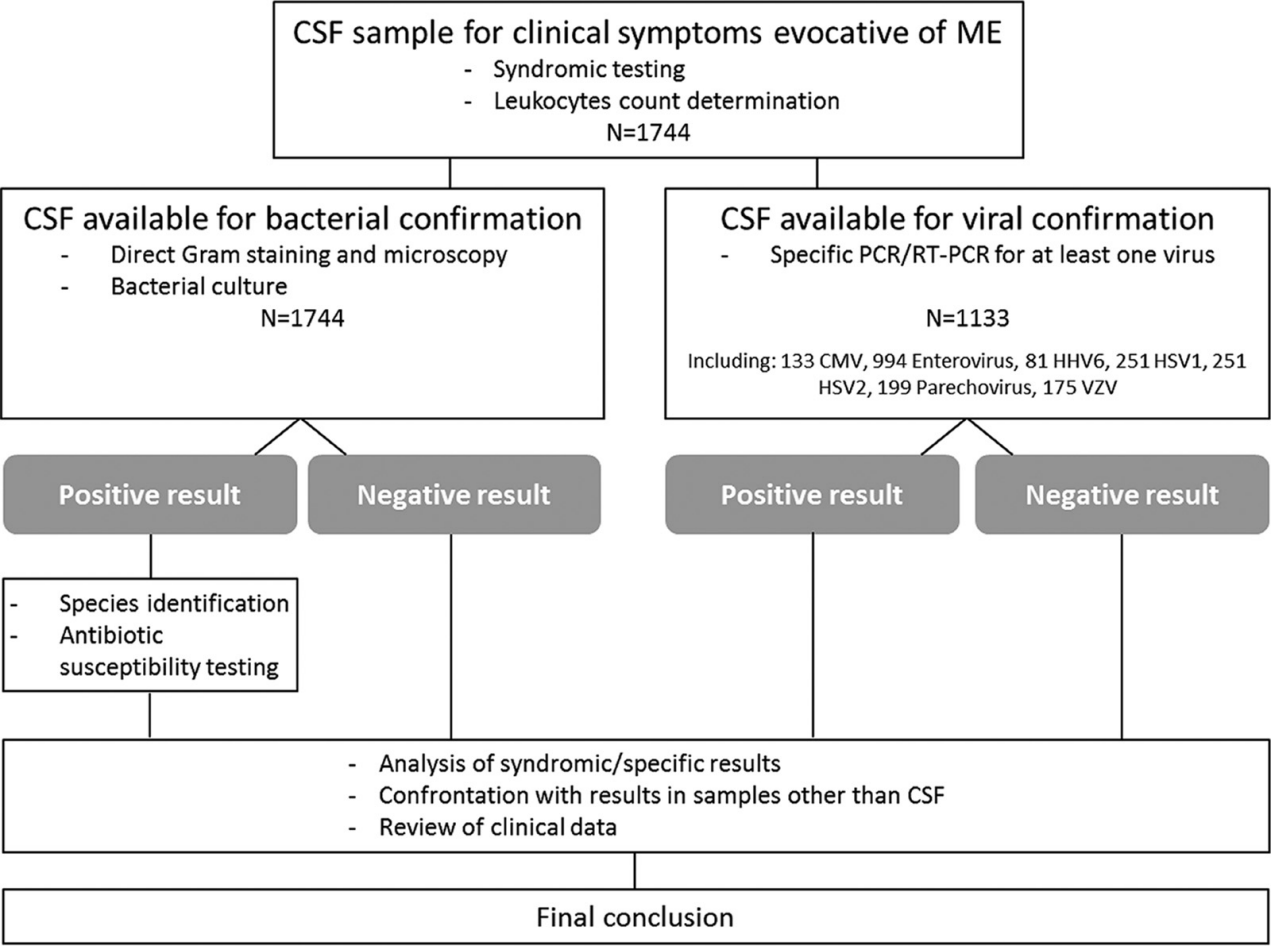
Flowchart of the study.

**FIG 2:**
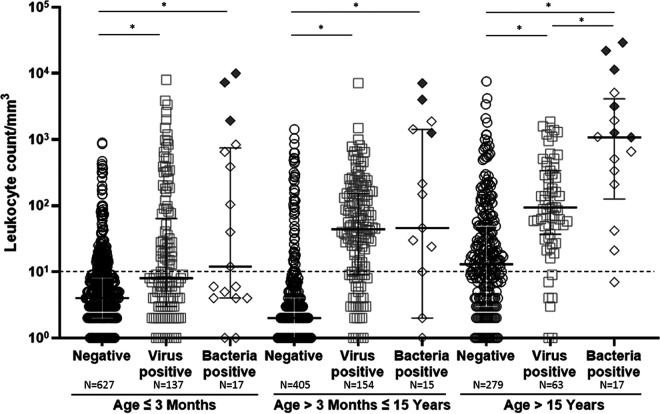
Leukocyte counts according to age group and positive detection for bacterial or viral targets. Gray diamonds indicate culture-confirmed samples. Bars show the median and the lower (Q1) and upper (Q3) quartiles. *, *P* < 0.05.

From May 2017 to November 2019, 1,744 CSF samples from 1,680 patients were analyzed, including those from 1,344 pediatric and 336 adult patients. Each sample was subjected to bacterial culture and a FilmArray assay, while at least one virus-specific PCR was available for 1,133 samples (Fig. 1). Cryptococcus neoformans*/gattii* results obtained with the FilmArray assay (all negative except for one culture-confirmed sample) were not delivered to the clinicians because of concerns concerning the reliability of the results (3). Overall, viral pathogens were detected in 361 (21%) CSF specimens and bacterial pathogens in 52 (3%), either with the FilmArray or using the comparative techniques. Coinfections were very rarely observed: five with viral and bacterial agents (one not confirmed by bacterial culture) and 10 with two distinct viral agents, most including human herpesvirus 6 (HHV-6) (*n* = 8). Among infants aged <3 months, the viral positivity rate was 17.7% and the bacterial positivity rate was 2.5% (Table 1); the median leukocyte count was 4 (range, 0 to 900), 8 (0 to 8,000), and 12 (0 to 10,000)/mm^3^ in the negative, virus-positive, and bacterium-positive subgroups, respectively (Fig. 2). Among children aged 3 months to 15 years, the viral positivity rate was 27.3% and the bacterial positivity rate was 2.6%; the median leukocyte count was 2 (0 to 1,430), 44 (0 to 7,100), and 46 (0 to 7,100)/mm^3^, respectively. Among older children and adults, the viral positivity rate was 17% and the bacterial positivity rate was 4.7%; the median leukocyte counts were 13 (0 to 7,600), 94 (1 to 1,870), and 1,080 (0 to 29,400)/mm^3^, respectively. In total, 803, 572, 430, 299, and 215 CSF samples had more than 5, 10, 20, 50, and 100 leukocytes/mm^3^, respectively, with an overall positivity of 265 (33.0%), 224 (39.2%), 201 (46.7%), 150 (50.2%), and 108 (50.2%) for viral pathogens and 38 (4.7%), 34 (5.9%), 33 (7.7%), 27 (9.0%), and 27 (12.6%) for bacterial pathogens, respectively.

**TABLE 1:** Population characteristics and pathogen detection^*a*^

Characteristic	Children		Adults, >15 yr
	≤3 mo	3 mo to ≤15 yr	
No. of CSF samples/1,744 (no. of patients/1,680)	810 (787)	576 (557)	358 (336)
Age (median)	26 days	4.1 yr	48.5 yr
Sex	495 M/315 F	317 M/259 F	192 M/166 F
CSF leukocyte count range (median)	0–10,000 (4)	0–1,880 (3)	0–7,300 (19)
Median CSF leukocyte count when >10/mm^3^ (no. of patients)	24 (*n* = 202)	83 (*n* = 92)	60 (*n* = 225)

Viral detection, no. (%/no. of patients)	143 (17.7%/142)	157 (27.3%/156)	61 (17.0%/61)
Comparator^*b*^/tested (no. false positive^*c*^)	130/133 (0)	146/150 (1)	21/22 (1)
FilmArray ME/tested (no. false positive^*c*^)	128/143 (1)	145/157 (0)	58/61 (1)
Positive detection with >10 leukocytes/mm^3^ (%)	61 (43%)	114 (73%)	55 (90%)

Bacterial detection, no. (%/no. of patients)	20 (2.5%/20)	15 (2.6%/14)	17 (4.7%/15)
Comparator^*b*^/tested (no. false positive^*c*^)	7/20 (4)	9/15 (6)	13/17 (4)
FilmArray ME/tested (no. false positive^*c*^)	16/20 (9)	9/15 (4)	11/17 (0)
Positive detection with >10 leukocytes/mm^3^ (%)	9 (45%)	11 (73%)	15 (88%)

a CSF, cerebrospinal fluid; M, male; F, female.

b Comparator = culture for bacteria, specific PCR for viruses.

c Criteria for conclusions concerning false-positive results are detailed in the text.

### Comparative analysis for viral pathogens.

False-positive results occurred only twice for the FilmArray (1 HSV-2, 1 varicella-zoster virus [VZV]) and twice for specific assays (1 EV, 1 HSV-1), whereas false-negative results occurred 30 times for the FilmArray (26 EV, 2 HSV-1, 1 human parechovirus 1 [HPEV-1], 1 VZV) and six times for specific assays (4 EV, 2 HPEV) (Tables 2 and 3). Overall, the positive predictive value (PPV) was ≥99% for all viruses by both techniques, except for HSV-1 by specific assay (83.3%) and HSV-2 and VZV by FilmArray (87.5% and 88.9%, respectively) (Table 2). The negative predictive value (NPV) was ≥99% for all viruses by both techniques, except for EV by FilmArray, reaching only 96.6%.

**TABLE 2:** Performance summary of the FilmArray ME assay versus comparator assays (virus-specific PCR or bacterial culture) after evaluation^*a*^

Pathogen	No. of detections		Sensitivity		Specificity		PPA		NPA	
	Comparator/no. tested	FilmArray/no. tested (no. unconfirmed^*b*^)	Comparator, TP/(TP + FN) (%)	FilmArray, TP/(TP + FN) (%)	Comparator, TN/(TN + FP) (%)	FilmArray, TN/(TN + FP) (%)	Comparator, TP/(TP + FP) (%)	FilmArray, TP/(TP + FP) (%)	Comparator, TN/(TN + FN) (%)	FilmArray, TN/(TN + FN) (%)
CMV	1/133	5/1,744 (3)	1/1 (100)	2/2 (100)	130/130 (100)	130/130 (100)	1/1 (100)	2/2 (100)	130/130 (100)	130/130 (100)
Enterovirus	250/994	251/1,744 (25)	249/253 (98.4)	226/252 (89.7)	741/742 (99.9)	741/741 (100)	249/250 (99.6)	226/226 (100)	741/745 (99.5)	741/767 (96.6)
HHV-6	9/81	29/1,744 (18)	9/9 (100)	11/11 (100)	70/70 (100)	70/70 (100)	9/9 (100)	11/11 (100)	70/70 (100)	70/70 (100)
HSV-1	6/251	4/1,744 (2)	5/5 (100)	3/4 (75)	246/247 (99.6)	246/246 (100)	5/6 (83.3)	3/3 (100)	246/246 (100)	246/247 (99.6)
HSV-2	7/251	9/1,744 (1)	7/7 (100)	7/7 (100)	244/244 (100)	244/245 (99.6)	7/7 (100)	7/8 (87.5)	244/244 (100)	244/244 (100)
Parechovirus	12/199	13/1,744 (0)	12/14 (85.7)	13/14 (92.9)	185/185 (100)	185/185 (100)	12/12 (100)	13/13 (100)	185/187 (98.9)	185/186 (99.5)
VZV	9/175	24/1,744 (15)	9/9 (100)	8/9 (88.9)	166/166 (100)	166/167 (99.4)	9/9 (100)	8/9 (88.9)	166/166 (100)	166/167 (99.4)
E. coli	1/1,744	3/1,744	1/2 (50)	2/2 (100)	1,741/1,741 (100)	1,741/1,741 (100)	1/1 (100)	2/2 (100)	1,742/1,743 (99.9)	1,742/1,742 (100)
H. influenzae	0/1,744	9/1,744	0/0	0/0	1,744/1,744 (100)	1,735/1,744 (99.5)	0/0	0/9 (0)	1,744/1,744 (100)	1,735/1,735 (100)
L. monocytogenes	0/1,744	1/1,744	0/1	1/1 (100)	1,743/1,743 (100)	1,743/1,743 (100)	0/0	1/1 (100)	1,743/1,744 (99.9)	1,743/1,743 (100)
N. meningitidis	2/1,744	4/1,744	2/4 (50)	4/4 (100)	1,740/1,740 (100)	1,740/1,740 (100)	2/2 (100)	4/4 (100)	1,740/1,742 (99.9)	1,740/1,740 (100)
S. agalactiae	2/1,744	3/1,744	2/3 (66.7)	3/3 (100)	1,741/1,741 (100)	1,741/1,741 (100)	2/2 (100)	3/3 (100)	1,741/1,742 (99.9)	1,741/1,741 (100)
S. pneumoniae	7/1,744	16/1,744	7/12 (58.3)	12/12 (100)	1,732/1,732 (100)	1,728/1,732 (99.8)	7/7 (100)	12/16 (75)	1,732/1,737 (99.7)	1,728/1,728 (100)

a PPA, positive percent agreement; NPA, negative percent agreement; TP, true positive; TN, true negative; FP, false positive; FN, false negative. Note that inconclusive results are not presented; data on pathogens not included in the FilmArray panel are not presented.

b FilmArray positive and not tested by the comparative assays.

**TABLE 3:** Analysis of positive results

Pathogen	Total no.	No. of detections		
		FilmArray inconclusive	FilmArray correct	FilmArray incorrect
FilmArray positive/comparator positive				
CMV	0			
Enterovirus	222		222	
HHV-6	9		9	
HSV-1	3		3	
HSV-2	7		7	
Parechovirus	11		11	
VZV	8		8	
Total virus	260		260	
E. coli	1		1	
H. influenzae	0			
L. monocytogenes	0			
N. meningitidis	2		2	
S. agalactiae	2		2	
S. pneumoniae	7		7	
Total bacteria	12		12	

FilmArray positive/comparator negative				
CMV	2	2		
Enterovirus	4		4	
HHV-6	2	2		
HSV-1	0			
HSV-2	1			1
Parechovirus	2		2	
VZV	1			1
Total virus	12	4	6	2
E. coli	2	1	1	
H. influenzae	9			9
L. monocytogenes	1		1	
N. meningitidis	2		2	
S. agalactiae	1		1	
S. pneumoniae	9		5	4
Total bacteria	24	1	10^*a*^	13

FilmArray negative/comparator positive				
CMV	1	1		
Enterovirus	27	1		26
HHV-6	0			
HSV-1	3	1	1	1
HSV-2	0			
Parechovirus	1			1
VZV	1			1
Total virus	33	3	1	29
*S. haemolyticus*	1	1		
S. aureus	1	1		
K. pneumoniae	1	1		
E. cloacae	1	1		
Contaminations	12^*b*^	12		
Total bacteria	16	16^*c*^		

a All samples were taken from patients under antibiotic therapy.

b Nine positive for coagulase-negative staphylococci or *Micrococcus* species, 2 for polymicrobial flora, and 1 for S. oralis.

c Bacteria not included in the FilmArray panel.

### (i) Concordant results (*n* = 260).

EV (*n* = 222) was the most frequently identified pathogen in our study, mainly in the pediatric population and associated with clinical symptoms of benign meningitis. As expected, HSV-1 detection (*n* = 3) was associated with encephalitis or neonatal infection and HSV-2 detection (*n* = 7) was associated with meningitis. All 11 cases of parechovirus infection occurred in neonates, and the presence of parechovirus was confirmed in other compartments, such as blood or stool, by the specific assay.

### (ii) Discordant results (*n* = 45).

For details on the discordant results, see the supplemental material. For seven discrepant cases, the FilmArray result was considered correct: four EV and two parechovirus false negatives by the specific assays, with the virus found in samples other than CSF, and one HSV-1 likely to be a false positive by the specific PCR. For 31 discrepant cases, the FilmArray result was considered incorrect: one false positive each for HSV-2 and VZV and false negatives for 26 cases of EV and one each of HSV-1, parechovirus, and VZV. For seven discrepant cases, the interpretation of the results was inconclusive: one case of EV and one of HSV-1 by specific assay only, two of HHV-6 by FilmArray only, and three of cytomegalovirus (CMV)—two by FilmArray only and one by specific assay only.

### Comparative analysis for bacterial pathogens.

Without considering CSF leukocyte counts, the PPV for global bacterial detection was 58% by FilmArray and 48% by culture. However, considering a threshold of 10/mm^3^ leukocyte count, the PPV reached 87% for the FilmArray and 58% for culture (of note, 17 positive culture results reported bacteria that are not included in the FilmArray panel, mostly contaminants, except for one true case of bacterial meningitis). The NPV was 99% for bacterial detection by both FilmArray and culture. Concerning the pathogens included in the FilmArray panel only, false-negative results were assigned in 10 cases by culture and none by FilmArray; false-positive results occurred in 13 cases by FilmArray and none by culture (Tables 2 and 3).

### (i) Concordant results (*n* = 12).

Twelve CSF samples were positive by FilmArray and had a concordant positive bacteriological culture (six pediatric and six adult patients): seven for Streptococcus pneumoniae, two for Neisseria meningitidis, two for Streptococcus agalactiae, and one for Escherichia coli K1. All had a CSF leukocyte count of >1,000/mm^3^ with a median of 5,550 and a range of 1,260 to 29,400 (Fig. 2).

### (ii) Discordant results (*n* = 40).

For 11 discrepant cases, the FilmArray result was considered correct: 10 CSF samples were “false negative” by culture but coherent with the FilmArray results because they were sampled after the beginning of antibiotic therapy: five cases of S. pneumoniae, two of N. meningitidis, and one each of S. agalactiae, E. coli K1, and Listeria monocytogenes, shown by FilmArray and not by culture, except for one sample that was positive for S. pneumoniae by FilmArray and Micrococcus luteus by culture after enrichment, finally classified as contamination. The median CSF leukocyte count for samples under antibiotic therapy was 877 (range, 40 to 5,100; one hemorrhagic). One discrepant CSF sample was positive for E. coli K1 by FilmArray but not by culture in the context of pyelonephritis in a 2-month-old patient, with a leukocyte count of 4/mm^3^, underscoring the ability of the test to detect small amounts of bacterial genome, including from potential blood contamination.

For 13 other discrepant cases, the FilmArray result was considered to be false: nine positive for Haemophilus influenzae and four positive for S. pneumoniae and considered to be contamination after considering the clinical data. Nine of the CSF samples had <10 leukocytes/mm^3^, one was coagulated, and the last three were coinfected either by an EV (*n* = 2) or by *Candida* (*n* = 1) (leukocyte counts of 388, 838, and 104/mm^3^, respectively). All 13 patients presented a favorable clinical outcome with (*n* = 10) or without stopping antibiotic treatment early (24 to 48 h, at first culture results).

The last 16 discordant CSF samples concerned pathogens not included in the FilmArray panel. The final conclusion was culture contamination for 12 FilmArray-negative samples. Indeed, nine were positive for coagulase-negative staphylococci or *Micrococcus* species, two with polymicrobial flora, and one with Streptococcus oralis. Of note, six of the of them had very low leukocyte counts (median, 8.5 [range, 0 to 21]). Five others showed a viral coinfection (4 EV and 1 HSV-2) and a leukocyte count between 24 and 210/mm^3^ (median, 46), and the patients received no antibiotic therapy. Among the *Micrococcus* species-positive CSF samples by culture, only one showed notable pleocytosis (1,430 leukocytes/mm^3^), but the inflammatory reaction was explained by Kawasaki illness. Finally, genuine pathogens were identified in four CSF samples by culture. Three were contaminated by blood in the context of endocarditis, pyelonephritis, or septicemia (range of leukocyte count from 1 to 42/mm^3^) and were positive for Staphylococcus aureus, Klebsiella pneumoniae, and Enterobacter cloacae, respectively. The last CSF sample was positive for Staphylococcus haemolyticus in the context of intrathecal chemotherapy with an infected catheter (leukocyte count, 510/mm^3^).

## DISCUSSION

Syndromic panels have been developed over the last decade and have become a component of the standard-of-care testing in a number of medical institutions for respiratory, gastrointestinal, or meningitis-encephalitis pathogens. Such syndromic approaches offer the perspective of a comprehensive result for the most frequent pathogens with a very limited time to the result. However, the true performance of these assays is yet to be evaluated, especially in pediatric and adult cohorts, independently of the manufacturer (2).

Our study reviews a 2.5-year period of implementation of the syndromic ME assay, mainly in a pediatric population, without exclusion criteria, for patients attending an emergency department in our hospital group. Overall, the positivity rate never exceeded 30% for any age group, even though the syndromic assay is designed to cover the main ME etiologies. Although steadily progressing due to technical improvements, the identification of pathogens responsible for ME is still challenging. One explanation for our limited positivity rate could be the lack of restriction of indications in our population. However, in a study on patients selected for aseptic meningitis based on clinical criteria, most cases (60%) also showed no identifying pathogen (4). Many obstacles can stand in the way of identifying an etiological agent for ME, including physiological processes, immune neutralization, and postinfectious mechanisms, as well as technical issues of a restricted scope or insufficient performance of the assays used. Numerous studies underscore the danger of considering syndromic assays as definitive, with the risk of precluding the additional search for a satisfactory diagnosis in specific cases, such as for travelers, the immunosuppressed, and fragile patients or those hospitalized for a long time (2, 5–7). Furthermore, the analytical performance for pathogens included in the syndromic panel must reach a defined level. Our study addresses this particular point. Moreover, although the FilmArray assay is simple to perform, basic technical knowledge on molecular techniques is still necessary to limit any risk of contamination. An accurate examination and interpretation of the melting curves is also required, as a false-negative N. meningitidis result has been previously reported (8) and we report here a false-positive VZV result in this context.

Our study had several limitations, including an information bias: no standard reference group was constituted and the final interpretation of a false-positive or false-negative result was based on the author’s judgment in light of the clinical data. However, our results show differential performance for bacterial versus viral targets.

Discrepancies between the syndromic assay and specific assays were frequent for viral pathogens, and the conclusion was most often in favor of the specific techniques. As expected for viral infections, leukocyte counts were of limited use, as the complete absence of pleocytosis was often observed, particularly in young infants, likely reflecting the rapid medical care and lumbar puncture performed before the recruitment of immune cells, even limited, becomes detectable in the CSF. Our data on EV represent one of the largest series reported to date, and our comparison between FilmArray and specific PCR highlights an unequivocal lack of sensitivity of the FilmArray for CSF, thus limiting the scope of the result if negative. Moreover, blood and/or peripheral sample testing is recommended to improve the EV diagnosis and requires specific PCR, as samples other than CSF have not been validated for the FilmArray assay. Several reports have highlighted similar concerns related to the suboptimal sensitivity of the FilmArray for certain targets, including EV and HSV-1 (6, 9). Concerning HSV-1, the only viral cause of encephalitis in our study, the small number of positive patients in our population, as is often the case, was a pitfall for performance analysis. However, only three of six samples tested by both FilmArray and specific PCR were concordant, and one discrepancy was interpreted as a FilmArray false negative. A negative HSV-1 result by FilmArray for a primary lumbar puncture and/or in cases of relevant clinical characteristics should thus never lead to the interruption of acyclovir treatment. Concerning HSV-2, VZV, and HPEV, the performance of the FilmArray assay appeared to be more reliable, but systematic HPEV detection, similarly to that of E. coli K1, is devoid of interest outside the neonatal period, as severe cases are extremely rare beyond the age of 3 months. Systematic detection becomes a true concern for CMV and HHV-6, generating for CMV additional potential anxiety-inducing tests for fear of a congenital infection and for HHV-6 the risk of misdiagnosis, which happened several times during our study period, resulting in prolonged hospitalization for surveillance or even unnecessary acyclovir treatment, including for a child with kidney failure, as already feared by other authors (10). Of note, an HHV-6 diagnosis can be considered only if blood is tested in parallel.

**FIG 3:**
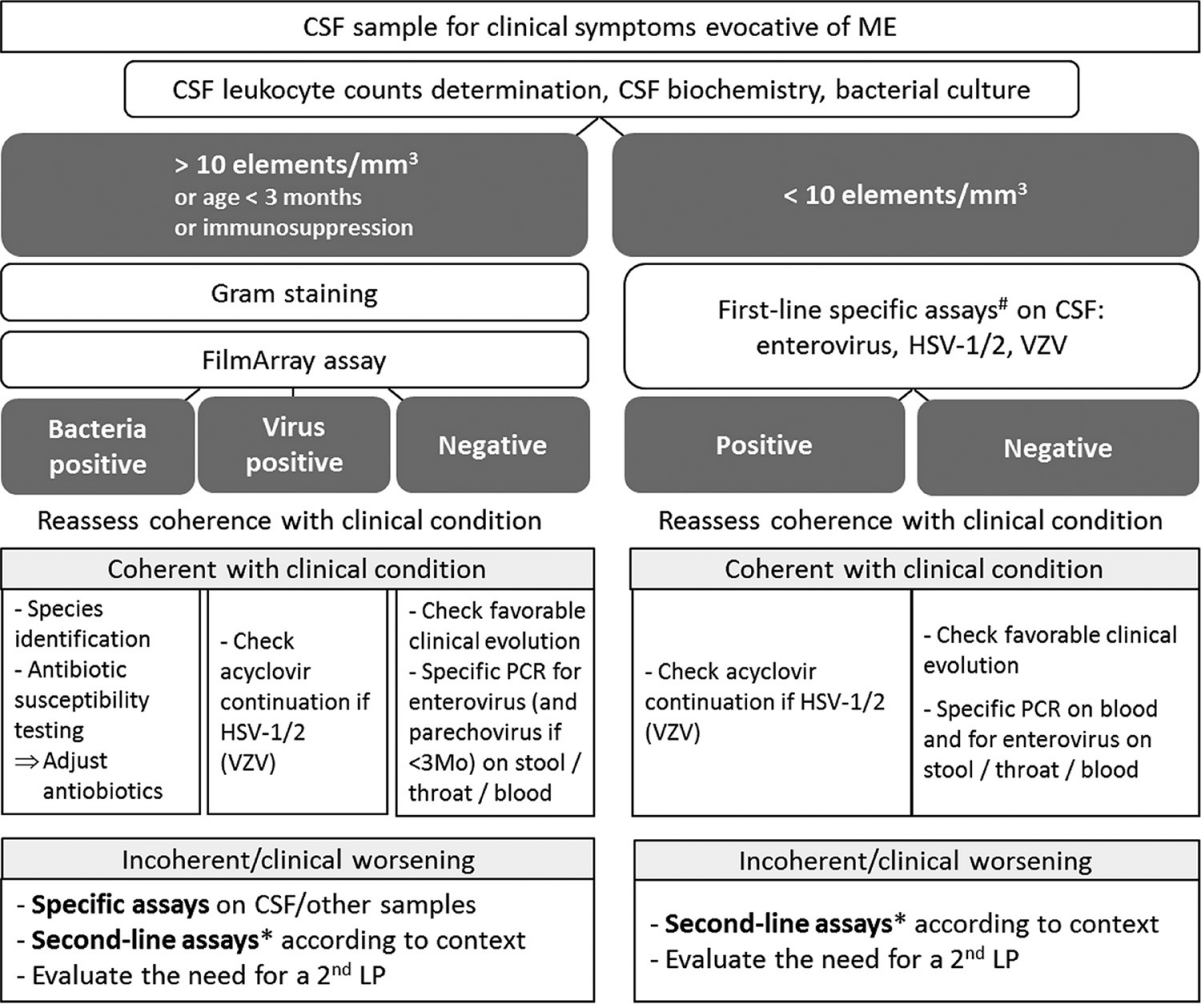
Algorithm for diagnostic assays to be performed according to the clinical situation. #, preferably by rapid specific PCR assays. *, nonexhaustive list: CMV, HHV-6, HIV serology, Epstein-Barr virus, adenovirus, West Nile virus, chikungunya virus, tick-borne encephalitis virus, JC virus, measles virus, etc.; intrathecal antiviral antibody detection, Mycoplasma pneumoniae, tuberculosis, syphilis and *Borrelia* serology, Chlamydia, *Bartonella*, *Coxiella*, etc.; and cryptococcal antigen, *Histoplasma*, etc. LP, lumbar puncture.

In terms of bacterial pathogens, we demonstrate the benefits of the wide use of the ME FilmArray, which can exceed the risk of false negatives, due to its good sensitivity, the short turnaround time compared to culture, the possibility of genome detection, even under antibiotic therapy, and the inherent risk of culture contamination. In our study, we mainly observed false-negative results in cases of the pathogen not being included in the panel of sought targets, highlighting the remaining position of bacterial culture as a gold standard. In cases of positive diagnosis, bacterial culture is obviously still necessary for antimicrobial susceptibility testing (11). The main issue for bacterial targets is still the low but significant rate of false-positive FilmArray results. As bacterial detection is a rare event, there is a true risk of a false positive in the presence of a positive result. Indeed, false-positive results, notably for S. pneumoniae, have been previously reported (9). In our study, all CSF samples were systematically tested with the FilmArray assay. Raising the threshold for systematic testing to 10 leucocytes/mm^3^ would have spared testing 1,092 CSF samples (−63%) to the detriment of 124 cases of positive viral detection and 15 of positive bacterial detection. Interestingly, the 124 cases of viral detection were better served by the specific assays, as 22 were FilmArray false negatives, and among the 15 cases of positive bacterial detection, 14 were FilmArray false positives and one was classified as inconclusive. Thus, the false-positive rate could be reduced by applying a sample-selection algorithm, as proposed in Fig. 3. The risk of overuse of the test has been previously mentioned, partially because of the rapid turnaround time; in a study for which there were only 6% positive results, the authors estimated that approximately one-quarter of the positive ME results were clinically insignificant (12). In another study, restricting testing to pleocytic CSF samples reduced ME panel utilization by 43% and increased the test yield by 62% (13). Péan de Ponfilly et al. showed that using a threshold of 10 leukocytes/mm^3^ reduced the rate of hospitalization, the length of stay, and empirical antiviral or antibacterial administration (14).

A crucial issue is whether implementing syndromic assays could help to lower the rate of unnecessary antibiotic treatment or at least promote strategies for their early discontinuation. In addition to participating in the environmental question of antibiotic resistance, this point would also define the “cost-effectiveness” of the use of the syndromic assay. This issue remains a complex and critical question. Nonetheless, this study constitutes a first step toward this goal for our hospitals. In light of the considerations listed above, we propose a patient-care algorithm to be applied in our tertiary university hospital structure to better address the place of the syndromic assay and increase its diagnostic yield, especially among our pediatric patients (Fig. 3). Our choice of a cutoff of 10 leukocytes/mm^3^ was made in an effort to reduce the risk of false-positive results by the syndromic assay, especially with H. influenzae and S. pneumoniae, both highly prevalent in this context and major pathogens. Indeed, no false positives remained for these two pathogens using this cutoff. Our results join those in a growing literature positioning the syndromic assay more as a screening test and not replacing conventional assays and recommending that it never be used as a “standalone” diagnostic option, especially in the presence of severe clinical presentations (2, 6, 9). We agree with the postulate that such a “simple” technique requires experienced users for accurate interpretation and discernment to properly interpret false-negative and false-positive results (6, 11). Further studies are warranted concerning feedback on the use and potential adaptation of this algorithm, which we first plan to apply to pediatric patients in our hospital to optimize the utilization of both syndromic and specific assays.

## MATERIALS AND METHODS

For this study, we analyzed comparative data obtained using the FilmArray ME panel (BioFire, Salt Lake City, UT, USA) versus those obtained using routine reference bacteriology and virology techniques, together with CSF cytology parameters and clinical data, over a 2.5-year period. CSF samples came from adult or pediatric patients with clinical symptoms evocative of ME presenting to an emergency department in our hospital group of eastern Paris, France. All assays were performed on standard-of-care samples. The pathogens included in the FilmArray assay are cytomegalovirus (CMV), EV, HSV-1 and -2, human herpesvirus 6 (HHV-6), human parechovirus (HPEV), varicella-zoster virus (VZV), Escherichia coli K1, Haemophilus influenzae, Listeria monocytogenes, Neisseria meningitidis, Streptococcus agalactiae, Streptococcus pneumoniae, and Cryptococcus neoformans*/gattii.* A critical analysis was performed considering the specific features of infection by each pathogen, with a special emphasis on pediatric versus adult cases.

### CSF sample management.

According to the organization of our laboratories, since May 2017, CSF samples have been processed in the two emergency laboratories (the Saint-Antoine site for adult samples from the Tenon and Saint-Antoine hospitals and the Armand Trousseau site for pediatric samples) 24 h a day, 7 days a week, for syndromic testing and CSF biochemistry parameters, whereas cytology parameters have been assessed during the night in the emergency laboratories and during the day in the centralized bacteriology laboratory; from June 2019 a second syndromic PCR device was acquired by the centralized bacteriology laboratory and has been used for adult CSF samples during the day. This retrospective study ended in November 2019. All patients who had their CSF tested using a FilmArray ME panel were included, with or without additional specific assays performed on the same CSF sample. Pediatric patients were under 16 years of age.

### Comparative techniques.

Syndromic testing was performed during the study period with the ME FilmArray assay in accordance with the manufacturer’s instructions. Routine virology was based on specific PCR or reverse transcription-PCR (RT-PCR) assays and was carried out in the routine virology laboratory of each site (Saint-Antoine, Tenon, and Trousseau), essentially systematically for pediatric patients and EV/parechovirus detection and on demand from clinicians for other viruses and for adult patients. Depending on the site and period of the study, specific viral PCR was performed using R-Gene assays (bioMérieux, Marcy l’Etoile, France) for HSV-1 and -2, VZV, CMV, EV, and HPEV; RealStar assays (Altona, Hamburg, Germany) for HSV-1 and -2 and VZV; Artus assays (Qiagen, Hilden, Germany) for CMV and HHV-6 A and B; Abbott RealTime assays (Abbott Molecular, Chicago, IL, USA) for CMV; Simplexa HSV-1 and -2 direct kit (DiaSorin Molecular, Cypress, CA, USA) for HSV; Xpert assays (Cepheid, Sunnyvale, CA, USA) for EV; and in-house assays for EV and HPEV. Regular comparative analysis between specific assays showed equivalence for clinical samples; equivalent performance was also routinely verified on quality control panels. Routine bacteriology was based on culture techniques and was systematically applied to all CSF samples during the study period and carried out according to the French recommendations (15). A sterile pipette was used to inoculate blood and chocolate agar plates and to prepare a Kovaslide chamber for cytological determination. Two drops of the CSF was centrifuged for 8 min at 50 × *g* to prepare for Gram staining. Broth enrichment could also be carried out in cases of ≥10 leukocytes/mm^3^ for patients <3 months of age and ≥5 leukocytes for patients >3 months of age. The agar plates were cultured aerobically at 35 to 37°C in a 5% CO_2_ atmosphere. Plates were examined after 24 h and then daily for a total of 5 days. Broth was cultured aerobically and examined daily for 7 days. Culture-based identification was performed by matrix-assisted laser desorption ionization–time of flight (MALDI-TOF) mass spectrometry (Bruker Biotyper; Bruker Daltonics, Billerica, MA). In cases of discrepancy, a FilmArray “correct,” “incorrect,” or “inconclusive” value was given based on the clinical data, leukocyte count, and antibiotic therapy initiated before sampling. Each sample with a culture positive for organisms not included in the FilmArray panel was considered to be “inconclusive.” A positive and negative predictive value was calculated for each method using the meningoencephalitis clinical status as a comparator.

### Statistical analysis.

Statistical analyses were conducted using GraphPad Prism v9 software (San Diego, CA, USA). Differences were evaluated using Mann-Whitney tests and considered to be significant for *P* values of <0.05.
