# Supplementary material for: Differential Performance of the FilmArray Meningitis/Encephalitis Assay To Detect Bacterial and Viral Pathogens in Both Pediatric and Adult Populations
Source: Microbiol Spectr. 2022 Apr 11;10(2):e02774-21. doi: 10.1128/spectrum.02774-21 (PMC9045182; doi:10.1128/spectrum.02774-21)
Supplement: SUPPLEMENTAL FILE 1 — Supplemental material. Download SPECTRUM02774-21_Supp_1_seq8.pdf, PDF file, 0.5 MB [file spectrum02774-21_supp_1_seq8.pdf]

Supplemental data

Details of the comparative analysis for discordant results

Viruses (n = 45):

For seven discrepant cases, the FilmArray result was considered to be correct:

- four enterovirus and two parechovirus false negatives by the specific assays, with virus shown in samples other than CSF, namely blood, stool, or throat swab
- one case of HSV1 likely to be a false positive by the specific PCR for a patient with multiple comorbidities, including acute myeloblastic leukemia, allografted five months earlier, suffering from GVHD, CMV reactivation, and probable invasive aspergillosis, for whom reversible neurological disorders were attributed to a cefepime overdose, before the patient died one month later from ARDS.

For 31 discrepant cases, the FilmArray result was considered incorrect:

- False positive for one case of HSV2 in an 87-year-old woman with post-stroke refractory epilepsy, negative in the blood and in a second lumbar puncture, who died three weeks later; and for one case of VZV, with abnormal melting curves for the FilmArray for a CSF sample from a three-month-old infant suffering from RSV bronchiolitis
- False negative for 26 cases of enterovirus: the specific PCR cycle threshold was higher for samples for which the virus was not detected *vs* those for which it was detected by the FilmArray (median Ct 39.49 vs 33.98, respectively, $p < 0.01$), reflecting a smaller amount of EV genome in the samples missed by the FilmArray
- False negative for one HSV1 sample from an eight-day-old infant with a neonatal HSV1, although detected postnatally, infection associated with evocative skin lesions and major hepatic cytolysis (the follow-up lumbar puncture was positive by both assays six days later)
- False negative for one case of parechovirus in a neonate with poorly tolerated fever and positive viral detection in samples other than CSF; and for one case of VZV in a 46-year-old woman with facial paralysis.

Among the seven discrepant cases for which the interpretation of the results was inconclusive, the patient for the one case of EV by specific assay only had a confirmed *S. pneumoniae* infection. The one case of HSV1 by specific assay only involved a 65-year-old patient with acute respiratory distress who was influenza A positive following the removal of a brain tumor and had an elevated blood HSV1 viral load. There was no concomitant blood sample for the two cases of HHV6 by FilmArray only. Among the three cases of CMV, two were by FilmArray only and required further investigations to identify a possible congenital infection (finally ruled out) and one was by specific assay only, for which the patient had an elevated blood CMV viral load.
